# Donor variability of ovine bone marrow derived mesenchymal stem cell - implications for cell therapy

**DOI:** 10.1080/23144599.2023.2197393

**Published:** 2023-04-17

**Authors:** E’atelaf A. Al-Mutheffer, Yvonne Reinwald, Alicia J. El Haj

**Affiliations:** a Institute for Science and Technology in Medicine, Keele University, Stoke-on-Trent, UK; b Department of Surgery and Obstetrics, College of Veterinary Medicine, Baghdad University, Baghdad, Iraq; c School of Science and Technology, Department of Engineering, Nottingham Trent University Nottingham, Nottingham, UK; d School of Chemical Engineering, Healthcare Technology Institute, Institute of Translational Medicine Birmingham University, Birmingham, UK

**Keywords:** Ovine bone marrow derived mesenchymal stem cells, donor variability, sheep model, cartilage repair, regenerative therapies

## Abstract

It is assumed that all species, including sheep, demonstrate significant variation between individuals including the characteristics of their bone marrow-derived mesenchymal stem cells (BM-MSCs). These differences may account for limited success in pre-clinical animal studies and may also impact on treatment strategies that are used within regenerative medicine. This study investigates variations between ovine MSCs (oMSCs) isolated from 13 English Mule sheep donors by studying cell viability, expansion, the cells’ trilineage differentiation potential and the expression of cell surface markers. In addition to the primary objective, this article also compares various differentiation media used for the trilineage differentiation of oMSCs. In this study, a clear individual variation between the sheep donors regarding oMSCs characterization, tri-lineage differentiation potential and marker expression was effectively demonstrated. The results set out to systematically explore the ovine mesenchymal stem cell population derived from multiple donors. With this information, it is possible to start addressing the issues of personalized approaches to regenerative therapies.

## 1. Introduction

Mesenchymal stem cells (MSCs) have been introduced as a possible cell source for orthopaedic tissue engineering due to their unique biological properties [1]). Several stem cell sources are under investigation, such as umbilical cord blood, amniotic fluid, bone marrow, and adipose tissue. However, clinical applications are mainly limited to bone marrow and peripheral blood-derived MSCs, which can be harvested easily and safely [2]. Animal models are commonly used method for testing medical substances or tissue engineering approach [3]. At present, animal models cannot give representative comparison for all research questions for orthopaedic regeneration in humans [4]. In fact, each animal model is selected to treat a specific research question. For instance, the mechanistic investigations of bone biology and response to growth factors could be assessed within small animals such as mice or rats. The repair of orthopaedic defects using engineered tissues and biomedical implants on the other hand must be studied in large animals, such as sheep, before their successful translation to human practice.

Sheep are commonly used for pre-clinical studies before clinical translation, since they are docile and their large bones have similar structure, biochemical, and mineral composition to humans’. In addition, the size and basic anatomy of the sheep skeleton and ageing are generally comparable with humans [5]. Hence, orthopaedic implants including engineered cartilage and bone tissues are commonly tested in sheep model and numerous studies have been performed [6–9].

However, there are some limitations associated with using sheep animal model for orthopaedic studies such as high cost, ethical consideration and quadrupedal gait [10]. Additionally, ovine MSCs (oMSC), unlike human MSCs (hMSC), are not well studied regarding their isolation, expansion, and characterization. Very few studies investigated the growth characteristics, differentiation, and surface antigen expression of oMSC [11]. Same as humans, there are individual variations between different sheep donors [12]. However, despite large donor-dependent variations, standard protocols and media compositions for human MSCs differentiation were first established by Pittenger in 1999 [13]. In contrast, oMSCs differentiation and characterization protocols are still lacking.

Articular cartilage (AC) is a specialized tissue which covers the articulating surfaces at the end of the mammalian bone. Cartilage is considered avascular, aneural, and has limited capacity for self-repair [14] due to its sparse cellularity, inactive appearance, and obscure characteristics [14–16]. However, researchers have defined not only the structural arrangement of the tissue and the complexity of the polydisperse matrix components but a surprisingly active set of metabolic processes [17].

This study aimed to determine variations between oMSCs obtained from 13 sheep donors to assess the potential influence of donor variations on clinical outcomes for regenerative medicine and cartilage tissue engineering. Therefore, cells were isolated and characterized, and cells’ proliferation and differentiation capacities were determined. Additionally, various media compositions were investigated for their suitability as trilineage differentiation media for the isolated oMSC. The isolated oMSC will be used to engineer donor-specific cartilage tissue.

## 2. Materials and methods

Materials were sourced from manufacturers or their distributors within the United Kingdom, unless otherwise mentioned (supplementary data SD-Table 1). Methods for all surgeries were performed in accordance with the UK Home Office Regulations and protocols confirmed by the University of Nottingham, Animal Welfare and Ethical Review Body. Thirteen adult female English Mule sheep, non-pregnant, skeletally mature (age 2–4 years) with weights ranging from 64.5 to 89.5 kg were used in this study. The sheep were housed at the Sutton Bonington Animal Facility at the University of Nottingham.

**Table 1.** Media used for the bone marrow collection, oMSC isolation and early expansion and proliferation. Basic medium was also used as control medium.

Media types	Compositions
Serum free media (SFM)	alpha minimum essential medium (αMEM) without L-glutamine; supplemented with 1% L-glutamine, 1% Penicillin/streptomycin (P/S)
Collecting media	alpha minimum essential medium (αMEM) without L-glutamine supplemented with 10% foetal bovine serum (FBS), 1% L-glutamine, 1% Penicillin/streptomycin (P/S), 1% heparin.
Early Proliferation Medium (EPM)	alpha minimum essential medium (αMEM) without L-glutamine supplemented with 20% foetal bovine serum (FBS), 1% L-glutamine, 1% Penicillin/streptomycin (P/S).
Basic Media (BM)	alpha minimum essential medium (αMEM) without L-glutamine supplemented with 10% foetal bovine serum (FBS), 1% L-glutamine, 1% Penicillin/streptomycin (P/S).

### 2.1. Isolation and expansion of oMscs

#### 2.1.1. Collection of ovine bone marrow

Under general anaesthesia, bone marrow was surgically aspirated from the sternum bone of the sheep using a sterile 50 ml syringe connected to a Jamshidi needle and coated with 1% heparin (Workhardt, UK). The aspirate was transferred to 50 ml falcon tubes containing collecting media (Table 1). Tubes were kept on ice for 1 h during the transport to the cell culture laboratory at Keele University where oMSCs isolation and Stro-4 selection were carried out.

#### 2.1.2. Isolation and stro-4 selection of oMscs by magnetic cell sorting (MACS)

All stock and working solutions were prepared for the isolation of bone marrow-derived ovine mesenchymal stem cells (BM-oMSC) and the Stro-4 selection process as described in SD-Table 2 (supplementary data). For each donor, the mononuclear cell fraction was isolated by red blood cell (RBC) lysis treatment. Stro-4 positive oMSCs were isolated by MACS as previously described by Markides et al. 2018 [18]. Cells were cultured with early proliferation medium (EPM) at 37°C and 5% CO_2_. After 3 days, non-adherent cells were removed, and basic medium (BM) was added. Cells were then cultured until 80–90% confluency and used for experiments at passage 3.

**Table 2.** Number of donors out of 13 that have expressed the CD makers. Scale was used to indicate the marker expression level as following −: no expression; ±: <5% expression; +: 5 − 50% expression, ++: 50 − 100% expression (27).

CD Expression	(-)no expression	(+ -) <5% expression	(+) 5 − 50% expression	(++) 50 − 100% expression	Total Donors
CD 29	/	/	4	9	13
CD44	/	/	1	12	13
CD 45	11	2	/	/	13
CD31	3	7	3	/	13

#### 2.1.3. Cell viability assessed by trypan blue exclusion test

The cell viability of isolated cells was assessed by trypan blue exclusion test [19]. Briefly, after trypsination and staining with trypan blue (Biosera, UK), the total numbers of live and dead cell were counted using a haemocytometer. Then, the number of cells per millilitre, the total cell number and cell viability were calculated.

#### 2.1.4. Assessment of metabolic activity

To assess cell viability, alamarBlue^TM^ reagent (Invitrogen, UK) [20] was used according to the manufacturer’s instructions. The assay was performed in triplicate on cell monolayers at passages (P) P1, P2 and P3. Following incubation, 100 µL of the assay solution from each well were transferred to a 96 well plate (*n* = 8). Acellular control samples (basic media) were used as blank. The absorbance was read at 570 nm, using 600 nm as a reference wavelength. Absorbance values were plotted as bar graph and standard deviations are shown as error bars.

### 2.2. Testing differentiation media for ovine BM-MSCs

Supplements required to prepare adipogenic media (AdM), osteogenic media (OsM) chondrogenic media (ChM) and other media used in this study were prepared as stock solutions, aliquoted and stored at −20°C until used. Various differentiation protocols were investigated to encourage oMSCs differentiation. Initially, two pilot studies were performed to test the media compositions’ suitability for the tri-lineage differentiation of the ovine BM-MSCs (supplementary data).

### 2.3. Tri-lineage differentiation of ovine BM-MSCs

From the pilot studies 1 and 2, media compositions for trilineage differentiation were chosen based on best performance for each lineage, namely for adipogenesis [21], for osteogenesis [22] and for chondrogenesis [23].

#### 2.3.1. Adipogenic differentiation of ovine BM-MSC monolayers

oMSCs of 13 donors were seeded as monolayers in 24 well plates at 2 × 10^4^ cells/well (*n* = 3). Cells were cultured using the induction and maintenance medium compositions described previously [24]. Briefly, cells were cultured initially with adipogenic induction medium consisting of high-glucose DMEM (4.5 g/L), 1 μM dexamethasone, 0.5 mM 3-isobutyl-1-methylxanthine (IBMX), 100 µM indomethacin, 10 µg/ml insulin, 1% non-essential amino acid (NEAA), 1% bovine serum albumin (BSA), 1% l-glutamine and 1% penicillin/streptomycin (P/S) for 72 hours. Then, medium was changed to adipogenic maintenance medium consisting of DMEM (4.5 g/L) + 1% l-glutamine, 1% BSA, 10 μg/ml insulin and 1% P/S for 14 days.

On day 1, day 7 and day 14 of incubation, cells were fixed with 10% buffered formalin. Adipogenesis of the monolayers was assessed by Oil Red O stain of intracellular lipid droplets. Therefore, a dye stock solution was prepared in 100% isopropanol. The stock solution was filtered and kept in RT until use. Prior to staining, a 60% (v/v) working solution was prepared in distilled water. This working solution was filtered using a 0.2 μm syringe filtered before use. Fixed cells were washed twice with distilled water before 500 μL of Oil Red O working solution were added to each well and incubated for 10 min at room temperature. Then, the dye was removed, and cells were washed four times with distilled water. Brightfield images were taken at different magnifications. The Oil Red O dye was eluted for semi-quantitative analysis. Thus, 200 µL of 100% isopropanol were added to each well, incubated for 10 min at room temperature. The absorbance of the eluted dye was measured at 492 nm (*n* = 6).

#### 2.3.2. Osteogenic differentiation of ovine BM-MSC monolayers

The human protocol for osteogenesis was used to compare the responses of ovine BM-MSCs obtained from 13 donors [24]. Thus, 2 × 10^5^ cells/well were seeded in 6 well plates (*n* = 3). At 90–100% confluency, cells were incubated with 4 ml of osteogenic media (OsM) containing DMEM (high glucose), 0.1 µM dexamethasone, 50 µM ascorbic acid, 50 mM β-glycerophosphate, 10% foetal bovine serum (FBS), 1% L-glutamine and 1% P/S. While the control plate was incubated with basic media (BM). Osteogenesis was evaluated histologically on day 1, 14 and 21 after cells were fixed using 95% methanol for 20 min. Cells were then stained with alizarin S red to determine the mineralized calcium deposits following osteogenic differentiation in the fixed monolayers. Therefore, 1% (w/v) alizarin S red dye was dissolved in dH_2_O. The solution was then filtered using a 0.2 μm syringe filter and the pH was adjusted to 4.0 using 0.1 M HCl. Fixed samples were washed with dH_2_O. Subsequently, the cells were fully covered with alizarin S red staining solution and incubated for 20 min at room temperature (RT). After that, the dye was removed, and the samples were washed with dH_2_O gently to remove excess staining solution. Samples were imaged under a light microscope. Semi-quantitative analysis was performed to assess osteogenesis by eluting the alizarin S red stain using 10% Cetylpyridinum chloride (CPC) for each well. Samples were incubated overnight at RT following which absorbance was read at 562 nm (*n* = 6).

#### 2.3.3. Chondrogenic differentiation of ovine BM-MSC in pellet culture

To compare the chondrogenic potential of ovine BM-MSCs, cell pellets were cultured as described by Jackson et al. [25]. Therefore, the chondrogenic media composition published by Heidari et al. [23] was utilized with slight modifications, namely DMEM (high glucose) containing 0.1 μM dexamethasone, 50 μg/ml ascorbic acid, 10 ng/mL TGF-β1, 50 µl/mL ITS, 1% FBS, 1% L-glutamine and 1% PS. Pellets were prepared using 5 × 10^5^ cells/pellet (*n* = 6). Pellets were harvested and stored at day 0, 14 and day 21. Chondrogenesis was assessed by histology using Alcian Blue stain and Glycosaminoglycans (GAGs) quantification using DMMB assay. The sGAG content was normalized to the DNA content of the same samples to determine the sGAG content in relation to cell number. The DNA content was assessed by PicoGreen assay following manufacturer’s instruction.

### 2.4. Expression of cell surface markers

The ovine BM-MSCs of the 13 donors were immunophenotyped by flow cytometry. Cells at passage 3 were cultured until 80% confluent and then labelled with CD29, CD44, CD45 and CD31 antibodies. In brief, cells were trypsinized, counted and resuspended to achieve a concentration of 5 × 10^6^ cells/ml in ice-cold blocking buffer (BB) [26] which was prepared by dissolving EDTA and bovine serum albumin in 500 ml PBS. Then, cell suspensions were incubated at 4°C for 1 h. Next, 1 ml aliquots of the cell suspensions were centrifuged at 300 g and 4°C for 10 minutes before the supernatants were discarded. Cells were incubated at 4°C for 15 minutes with 100 μL of primary antibody solution (1:1000). Subsequently, cells were washed with BB and centrifuged at 300 *g* for 10 min before supernatants were discarded. Next, FITC-conjugated secondary antibody (Goat Anti-Mouse IgG (FITC) Abcam (ab6785) was added to the cells (1: 100) followed by incubation in the dark at 4°C for 15 min. Subsequently, cells were washed with 1 ml BB, centrifuged at 300 *g* for 10 min and finally resuspend with 150 μL PBS.

For CD31, control isotypes IgG1 and IgG2α cell labelling was minimized to three steps. Namely, after resuspending with 100 μL cold BB, cells were mixed with 10 μL of relevant FITC-conjugated primary antibodies (1:10) and incubated in dark at 4°C for 10 min. Cells were then washed with 1 ml cold BB and centrifuged at 300 *g* for 10 minutes. Finally, cells were resuspended with 150 μL PBS.

The flow cytometer Cytomics FC 500 (Beckman Coulter) with at least 50,000 event counts was used for analysis. The acquired data were analysed using Flowing Software (version 2.5.1). IgG1 was the isotype control for CD 29, CD 44 and CD 45. While IgG2α was the isotype control for CD 31. The FL1 channel was used for analysis. CD29 and CD44 were positive MSC markers and CD45 and CD31 were negative MSC markers. The percentage of cells that were considered positively stained was determined by gating the stained population with a gate that excluded 99% of all isotype control events. The results were scored as recommended by Boxall and Jones (2012) who scored MSC marker expression levels using the same scale, namely −: no expression; ±: <5% expression; +: 5 − 50% expression, ++: 50 − 100% expression [27].

### 2.5. Statistical analysis

Values for trypan blue and alamar blue; semi-quantitative data for adipogenic and osteogenic differentiation, the pellets’ sGAG content and sGAG/DNA content were plotted as bar graphs. The data were expressed as mean ± standard deviations. One-way ANOVA with Tukey’s multiple comparisons test was performed to determine statistical significance using SPSS statistics program version 24. Statistical signiﬁcance was set to 0.05 and *p* values were denoted as *p < 0.05, **p < 0.01, ***p < 0.001.

### 2.6. Ethical statement

Methods were conducted in accordance with the UK Home Office Regulations and protocols approved by the University of Nottingham Animal Welfare and Ethical Review Body. For all surgeries, animals were placed in lateral recumbency to allow access to the sternum and medical aspect of both hind legs.

## 3. Results

### 3.1. Cell expansion and cell viability

**Figure 1.**
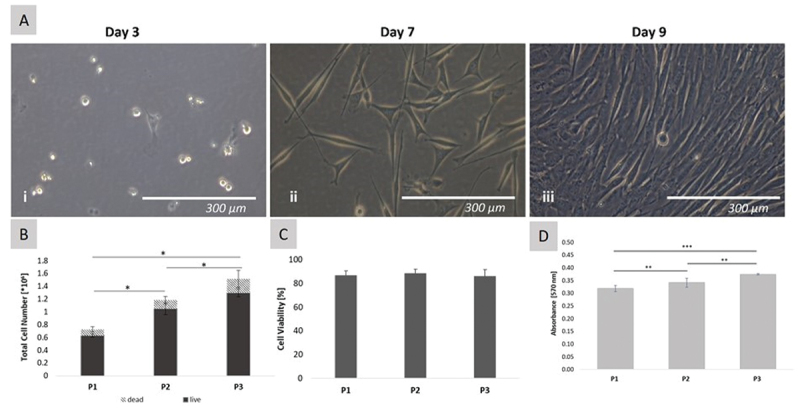
Characterization of isolated ovine BM-MSCs. A) Morphology of STRO-4 positive ovine BM-MSCs in 2D cell culture was observed at (i) 10% confluence on day 3, (ii) 50% confluence on day 7 and (iii) 90% confluence on day 9 after isolation. Cells exhibited characteristic spindle shape and polygonal morphology. B) Numbers of live and dead cells were counted using trypan blue for three passages (P1–3). C) Cell viability (%) was determined for one donor. Absorbance values were plotted as a bar graph. Standard deviations are shown as error bars. The data are expressed as mean ± standard deviation (*n* = 4, each). D) Metabolic activity of ovine BM-MSC at P1-P3 was assessed by alamarBlue^TM^. The results were normalized to an acellular blank and data are expressed as mean ± standard deviation (*n* = 8, each). *p ≤ 0.05 **p ≤ 0.01 ***p ≤ 0.001.

Cells adhered to stand tissue culture plastic three days after seeding (Figure 1A). Cells exhibited characteristic spindle shape and polygonal morphology on day 7 and 9 (Figure 1A). Cell viability was quantified for one donor over three passages using trypan blue exclusion test. The number of live cells increased significantly over three passages (*p* ≤ 0.001) from 0.628 × 10^6^ cells (±0.09 × 10^6^ cells) in passage 1 (P1) to about 1.047 × 10^6^ cells (±0.135×10^6^ cells) in passage 2 (P2) and 1.295 × 10^6^ cells (±0.22× 10^6^ cells) in passage 3 (P3). No significant differences in the number of dead cells were observed across the three passages indicating cells’ vitality and activity during the early passages. The values are expressed as total number of dead and live cells (Figure 1B). Cell viabilities of over 85% were observed for P1–3 and no significant differences between the three passages were observed (Figure 1C). Over three passages, metabolic activity was assessed by alamarBlue^TM^. Significant differences were observed in the metabolic activity of the cells (*p* ≤ 0.001). The absorbance of alamarBlue^TM^ increased from 0.32 nm ±0.012 (P1) to 0.33 nm ±0.026 (P2) and 0.38 nm ±0.003 (P3) (Figure 1D).

### 3.2. Adipogenic differentiation potential

**Figure 2.**
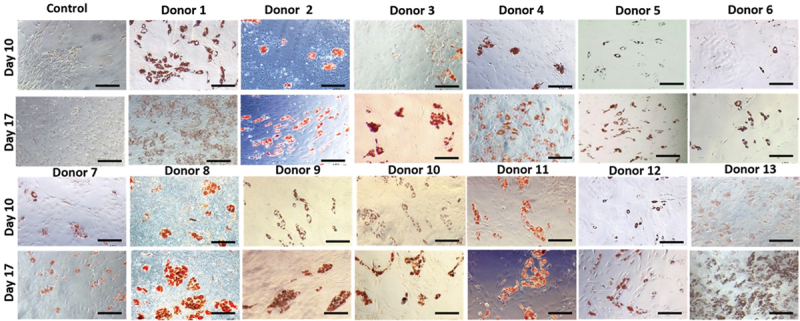
Oil Red-O stain of ovine BM-MSCs in monolayer. Bright field images of thirteen donors at P3 were taken on day 10 and day 17. Different degrees of adipogenic responses to the differentiation media were observed ranging from low (donor 5,6,9 and 12), moderate (donors 2,3,4,7 and 10) and high (donors 1,8,11,13). Scale bars = 300 µm.

**Figure 3.**
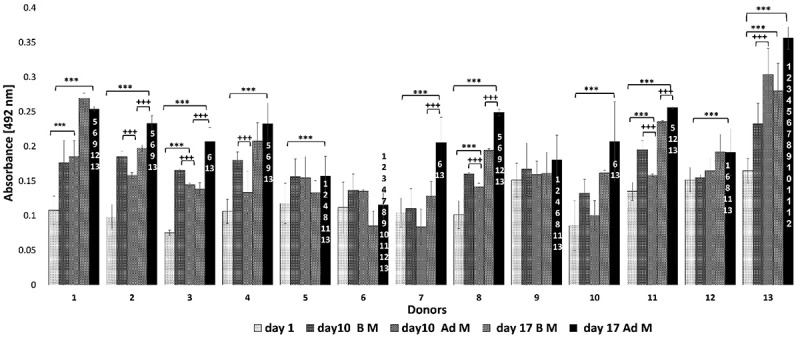
Semi-quantitative measurement of oMscs’ adipogenic differentiation. The measurement of the eluted dye revealed the occurrence of adipogenesis in the treated group (AdM) over 17 days. Significant differences were observed on day 10 and day 17 compared to day 1; except for donor 6 and 9. Significant differences were observed between individual donors indicating variations in adipogenic potential over 17 days. Figure specific symbols were given to compare the results as the following (+) comparison of significance levels between the treated (AdM) and control (BM) group of the same time points within the same donor; (*) comparison of significance between three time points for the treated group (AdM) for each donor; and the numbers [1–13] were used to compare adipogenesis between the thirteen donors at day 17 of the treated group, *p* ≤ 0.001.

Microscopic observation of Oil Red-O stained oMSC monolayers revealed that all donors underwent adipogenic differentiation at different levels (day 10, day 17) compared to the control groups which were cultured in basic medium (BM) (Figure 2) indicating variations in adipogenic potential. Hence, donors were divided into three groups, namely high performing donors (strong Oil Red O stain), medium performing donors (intermediate Oil Red O stain) and low performing donors (weak Oil Red O stain). This result was confirmed semi-quantitatively by spectrophotometry of the eluted dye (Figure 3).

In general, all donors showed significant differences across the duration of the experiment compared to day 1; except donor 6 and donor 9. Significant differences were also observed for most adipogenic differentiated cells compared to the controls (BM) at the same time points for each donor. However, when comparing all 13 donors with each other, noticeable differences were observed among donors regarding the final time point (day 17). These results confirmed that donors’ capacity for adipogenic differentiation was significantly different from each other, in particular donor 13 which showed the highest degree of the adipogenesis (absorbance 0.356 nm ±0.015). Donor 13 was significant different from all other donors (*p* ≤ 0.001). While the lowest degree of adipogenesis was observed for donor 6 (absorbance 0.115 nm ±0.022).

### 3.3. Osteogenic differentiation potential

**Figure 4.**
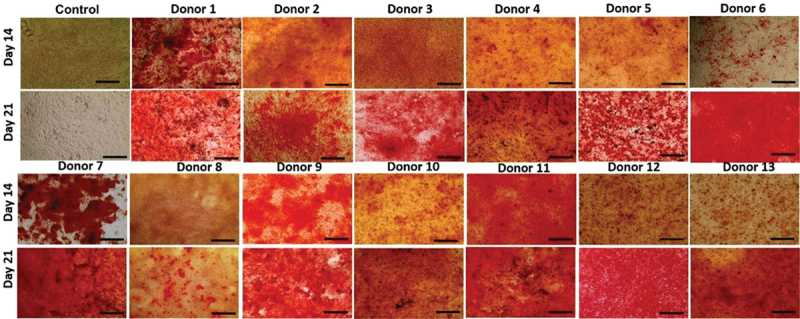
Alizarin Red S stain of oMscs. Bright field images of alizarin red stained oMscs (P3) in monolayers were taken on day 14 and day 21. Different degrees of calcium deposition were observed ranging from low (donors 3 and 8) to moderate (donors 2,4,6,9, 11, 12 and 13) and high (donors 1,5,7 and 10, 12) responses to the differentiation media. Images were taken at x10 magnification, scale bar = 300 µm.

oMSCs underwent osteogenic differentiation over 21 days. Calcium deposition was assessed qualitatively by alizarin red stain (Figure 4). Variations in the osteogenic differentiation potential were observed between donors when comparing 14 and day 21 to day 1 (*p* ≤ 0.001). The donors’ responses to differentiation media (OsM) ranged from low (donors 3 and 8) to moderate (donors 2, 4, 6, 9, 11, 12 and 13) to high (donors 1, 5, 7, 10 and 12).

**Figure 5.**
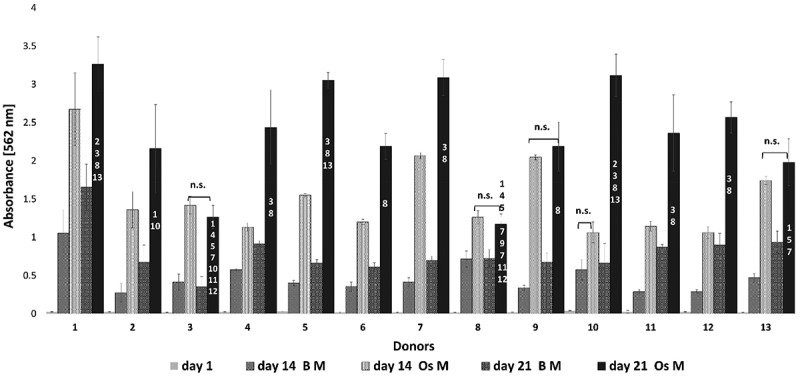
Semi-quantitative measurement of the oMscs’ osteogenic response. The absorbance measurement of the eluted Alizarin Red S revealed that the treated group (OsM) underwent osteogenic differentiation over 21 days. All donors showed significance differences at day 14 and day 21 compared to day 1. Treated groups (OsM) were significantly different from control groups (BM) at the same time point for each donor (*p* ≤ 0.001). Non-significant differences (n.S.) for all comparisons are shown. All other comparisons were significant at *p* ≤ 0.001. Numbers [1–13] represents the donor number in comparison to the other donors at the final time point, *p* ≤ 0.001.

This result was confirmed semi-quantitatively by eluting alizarin red (Figure 5). To allow easier comparison and understanding of the graph only non-significant (n.s.) differences are displayed, whereas significant differences were not displayed. For the comparison of treated (OsM) versus control (BM) cells at each time point for each donor; and the comparison between of the treated group on day 1, day 14 and day 21 for each donor, *p* ≤ 0.001 was assumed to be significantly different. The numbers [1–13] were used to illustrate the significant differences between treated donors on day 21. For each donor, significant differences in osteogenesis were observed for day 10 and day 21 compared to day 1 (*p* ≤ 0.001). The results also showed differences between the treated groups (OsM) compared to the control group (BM) for each time point and each donor. oMSCs from donors 8, 3, and 13 had the lowest osteogenic differentiation potential. Lastly, donors 5, 1, 10, and 7 could be considered as high osteogenic performers as illustrated by the highest absorbance readings.

### 3.4. Chondrogenic differentiation potential in pellet culture

**Figure 6.**
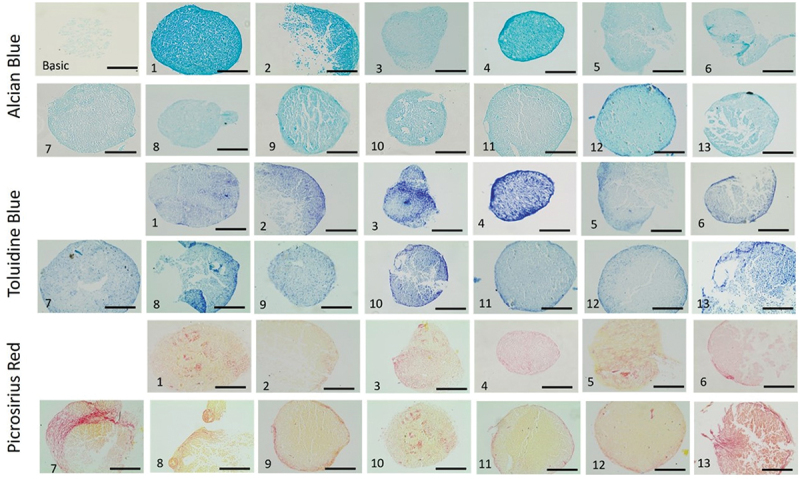
GAG and collagen production in ovine BM-MSCs pellets. Three different histological stains were performed on 7 µm thick paraffin sections of cell pellets cultured in ChM and BM on day 21. Variations in the production of GAG (Alcian blue and toluidine blue) and collagen (picrosirius red) were observed reflecting the chondrogenic potential of the thirteen donors. Considering GAG production, donors 1, 2, 4 and 12 were considered high performers, while the remaining donors which showed a lower degree of chondrogenesis. The highest amount of collagen was produced by donors 7 and 13 performed best. Images were taken at 20 × magnification, scale bar = 200 μm.

The chondrogenic differentiation potential of the oMSCs was assessed by histological staining which revealed chondrogenic differentiation for all 13 donors in the treated group (ChM) compared to the control group (BM) (Figure 6). Two histological stains for sGAG were used to assess matrix production, namely Alcian blue and toluidine blue. The strongest Alcian blue and toluidine blue stains were observed for donors 1, 2, 4, and 12. Collagen production was assessed by picrosirius red stain, which indicated the strongest stains for donors 7 and 13.

**Figure 7.**
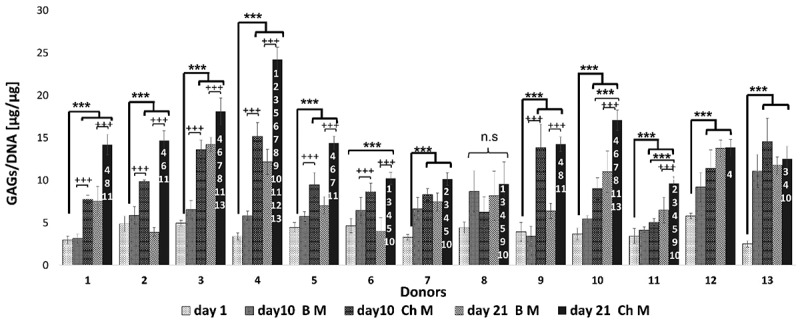
Production of GAG in relation to DNA content. The pellets’ GAG and DNA contents were quantified using DMMB and PicoGreen assay respectively. Significant increases were observed on day 10 and day 21 compared to day 1 for the treated group (ChM) for most donors. Donor 8 revealed no significant differences between day 10 and 21 compared with day 1. (+++) = significant differences between the treated (ChM) and control (BM) for each time point and donor, (***) = significant difference between the time points of Ch M for each donor at *p* ≤ 0.001 [1–13]. = significant differences in the comparison of each donor at day 21 for ChM with all other donors at the same time point. Each number [1–13] represents the donor number in comparison to the other donors at the final time point. Data are shown as standard deviation (*n* = 3), *p* ≤ 0.001.

Chondrogenesis was also assessed quantitatively by assessing sGAG contents in the pellets using DMMB assay. The sGAG production in the oMSCs pellets varied between donors. In general, the amount of sGAG per cell increased progressively until day 21 for all donors. The highest sGAG/DNA ratio was obtained for donor 4, whereas donor 12 showed the lowest sGAG/DNA ratio. The remaining donors have maintained their sGAG levels after normalization to the pellets’ DNA content (Figure 7).

**Figure 8.**
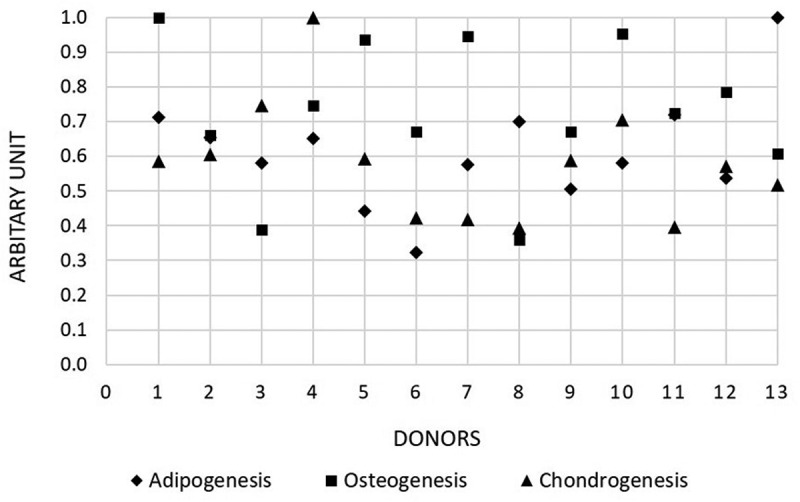
Comparison of donor differentiation. The donors’ differentiation performances were compared by calculating the differentiation as arbitrary unit. Therefore, highest absorbances for eluted alizarin red stain, Oil-red stain and GAG/DNA content on the final time point were set equal to 1 (donor 1, 4, 13). All absorbances were divided by the highest values and plotted as scatter plot.

The three differentiation lineages of each donor were compared using the absorbances of the eluted stains and GAG/DNA on the final time points, respectively. The highest values for adipogenesis (donor 13), osteogenesis (donor 1) and chondrogenesis (donor 4) were set to 1. The absorbances were normalized to the highest value and plotted as scatter graph (Figure 8). Although each donor’s oMSCs population differentiated, there was no clear profile in highly responsive donors between the three lineages. This means that a donor that was highly responsive during chondrogenic differentiation (donor 4), was not as responsive to osteogenic or adipogenic differentiation.

### 3.5. Surface epitope expression

**Figure 9.**
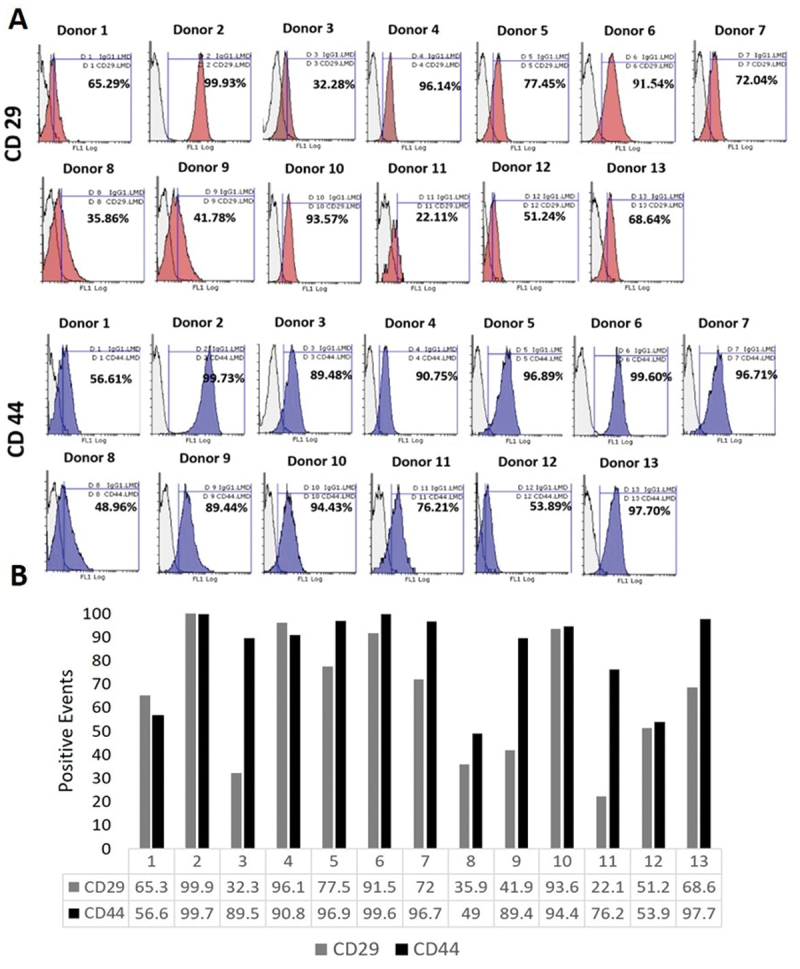
Immunophenotyping of oMscs for CD 29 and CD 44. Immunophenotyping of oMscs for CD 29 and CD 44. oMscs were cultured to P3 and 80% confluency in basic medium (B M). The expression of CD 29 and CD 44 was assessed to identify their MSCs characteristics. (A) Overlay histograms of each antibody marker, (B) positive cells (%) compared to staining with the IgG1 isotype control. Donors were positive for CD 29 and CD 44. Variations in the expression levels for both CD markers were obtained and ranged between 22.11% − 99.93% for CD 29 and between 48.96–99.73% for CD 44. The unfilled region is isotype control IgG1, red and blue regions are CD 29 and CD 44 antibody markers, respectively.

Flow cytometry of oMSCs was performed for four CD markers to identify oMSCs characteristics (Figure 9). The results showed that most donors were positive for both CD 29 and CD 44, however, differences were observed in the level of expression. CD 29 for example, was more strongly expressed in donor 2, donor 4, donor 6 and donor 10. While other donors demonstrated lower expression with less than 50% (donor 3, 8, 9, 11). The remaining donors showed mediocre positive expression (donor 1, 5, 7, 12, 13).

Regarding CD 44, higher positive expressions were observed compared to CD 29. Most donors were strongly positive for CD 44 (donor 2, 4, 5, 6, 7, 10, 13). While the remaining donors showed a mediocre positive expression (donor 3, 9, 11) or low positive expression (donor 1, 12, 8).

**Figure 10.**
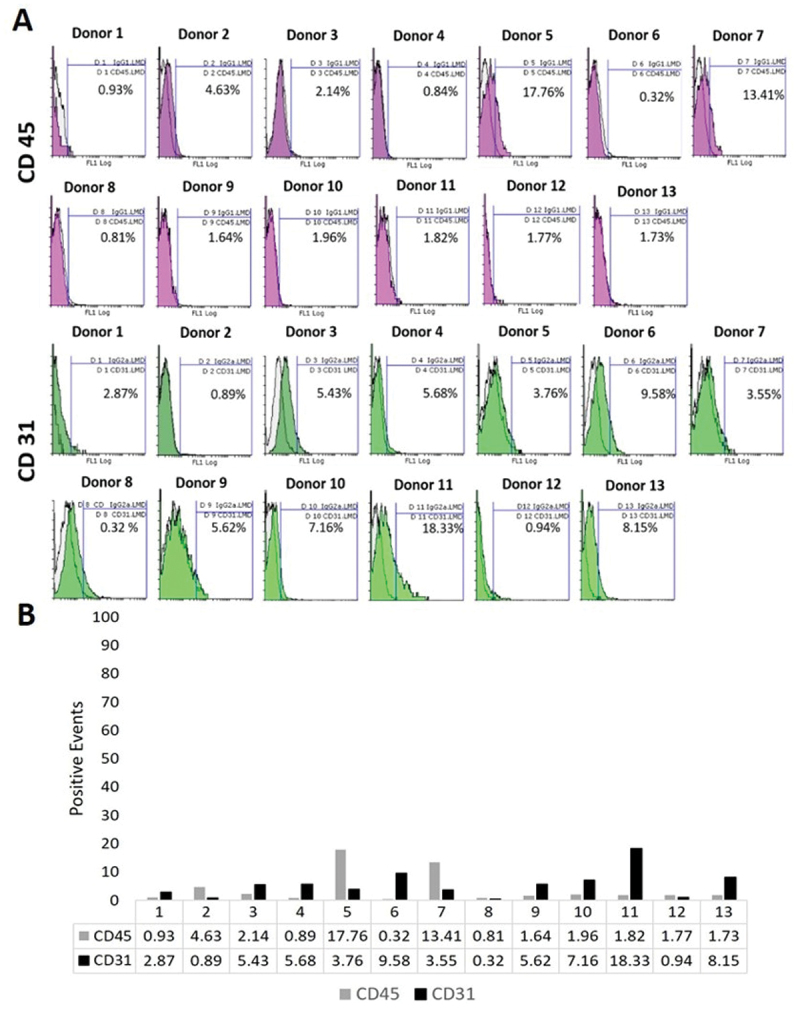
Immunophenotyping of oMscs for CD 45 and CD 31. oMscs were cultured until P3 and 80% confluency in basic medium (BM). The expression of CD 45 and CD 31 was assessed to identify their MSCs characteristics. (A) Overlay histograms of each antibody marker, (B) positive cells (%) compared IgG1 or IgG2α isotype controls. As expected, most donors were negative for both CD markers and showed variability in their expression levels, which ranged between 0.32% − 13.41% for CD 45 and between 0.32–18.33% for CD 31. Unfilled regions are isotype controls IgG1or IgG2α, purple and green regions are CD 45 and CD 31 antibody markers, respectively.

All donors showed low expression for both CD 45 and CD 31 (Figure 10), which is expected and is used to identify MSCs. For CD 45, most donors showed an expression between 0.32% and 4.63% except donor 5 and 7. For all donors, except donor 11, CD 31 was expressed at levels below 10%.

Results are shown in Table 2 using the recommended scale by Boxall 2012, which gave symbols indicating the marker expression level as following −: no expression; ±: <5% expression; +: 5 − 50% expression, ++: 50 − 100% expression.

## 4. Discussion

MSCs as therapeutic agents in the advancement of skeletal stem cell-based therapies have demonstrated remarkable clinical potential. Their restricted availability and difficult expansion to therapeutic numbers are still limiting their clinical use. MSCs represent less than 0.001% of the bone marrows’ cell population. Therefore, efforts to enrich their proportion are under development to harness their unique properties including the ability to self-regenerate, differentiate into several cell lineages and participate in immunomodulation. There is an increasing awareness of the MSCs’ various clinical applications in the treatment of many incurable diseases. Hence, their mechanisms of action, migration and potential individual variation among patients in the responses to the MSCs treatment as well as MSCs’ safety for clinical use of homologous treatments is of increasing interest.

For this study, ovine bone marrow derived MSCs were characterized to determine donor variation to aid the establishment of regenerative therapies and to predict their efficiency for clinical bone and cartilage repair.

All oMSCs investigated in this study were obtained from female sheep. Currently, there are no guidelines on the preferred use of male or female animals. However, Berset et al. [28] conducted a survey on Sheep Usage in Biomedical Research where sheep users were asked to confirm the sex of the animals they used for their studies. Results indicated that 79.8%, 19% and 16.7% of researchers used females, neutered males and intact males, respectively. For the 19% of researchers using neutered males, either the sex of the animals was not important, or animals were selected based on availability. Other important points were sheep availability and the trust into and experience of the sheep supplier [28].

oMSCs were first isolated from bone marrow in 1994 by Jessop and colleagues. They emphasized that cells exhibited a fibroblastic morphology and could be induced into adipogenic and osteogenic phenotypes *in vitro* [29]. Since then, oMSCs have been effectively isolated from different sources like umbilical cord blood [30], adipose tissue [31], peripheral blood [32], liver [23], amniotic fluid [33], dental pulp [34], synovial membrane [35], dermis [36], hair follicles [37] and endometrium [38].

There are several sites for collection of bone marrow from sheep. In the majority of sheep studies, the iliac bone is the preferred site for harvesting [11,26,39]. However, for this study, the bone marrow aspirate was successfully collected from the sternum bone and adequate amounts of bone marrow was harvested as described elsewhere [40,41]. Human MSCs (hMSC) can also be collected by bone marrow aspiration from the iliac crest of healthy volunteers [42]. While in infants and toddlers, the anteromedial face of the tibia is ideal. All studies showed that ovine MSCs exhibited morphological, immunophenotypical and multipotential characteristics similar to those observed in human MSC *in vitro* and *in vivo* [23,26,39].

In this study, oMSCs successfully adhered to the surface of standard tissue culture formats within 3 days of incubation. The cells displayed fibroblast-like morphology with spindle or triangular-shaped cell bodies, with large and elliptical nuclei. Cells proliferated in a fibroblast-like pattern. Similar observations were made previously [23]. Since oMSC are smaller than the hMSC [11], more ovine MSC could proliferate on the same culture area.

STRO-1 is a well-considered cell surface antigen employed for characterization of human MSCs populations [43]. Oreffo and colleagues have shown that through STRO-1 selection, it is possible to enrich the MSC population during cell isolation [44]. Gronthos and colleagues have developed and characterized the analogous ovine marker, STRO-4. Hence, STRO-4 positive oMSC were selected for this study because they exhibit multilineage differentiation potential capable of forming mineralized bone matrix, lipid-filled adipocytes, and chondrocytes capable of forming a glycosaminoglycan-rich cartilage matrix [45].

Passaging of oMSCs did not cause changes to their cell viability, cell morphology and cell characteristics typical for MSC, similar to other studies [32,46]. No significant changes were observed for cell viability obtained from trypan blue exclusion test. Nevertheless, significant differences were detected for cell metabolic activity evaluated by Alamar blue. This could be due to the nature of the different assays as trypan blue depends on the number of live and dead cells which increased significantly and gradually without affecting the viability percentage. On the other hand, Alamar blue usesuses the reducing capacity of living cells to quantitatively determine cell metabolic activity. When cells are alive, they maintain a reducing environment within the cytosol of the cell. In this study, the oMSCs remained healthy and viable over the three passages.

The isolated cells were evaluated for their three-lineage differentiation potential after appropriate differentiation media compositions were selected according to the histological performance of the seeded cells (supplementary data). The results for the pilot study 1 when applying either a standard hMSCs protocol or the recommended oMSCs protocol for differentiation, both protocols resulting in osteogenesis, while limited adipogenesis and chondrogenesis were observed. Therefore, the protocol by Jaiswal et al. 1997 was chosen for osteogenic differentiation [24]. Additionally, different protocols were tested on two oMSC donors to determine appropriate media composition for adipogenesis [13,21,23,47,48] and chondrogenesis [21,23,26,47,48] (pilot study 2). In this study, the adipogenesis and chondrogenesis protocols established by Heidari et al. 2013 were selected for all following differentiation experiments [23].

For the differentiation media, all 13 donors exhibited positive osteogenic, adipogenic and chondrogenic differentiation compared to the basal (BM) which contains the essential elements for proliferation and expansion and was used as control. Clear variations between donors were observed through histological stains and their semiquantitative analysis as well as biochemical assays either in 2D or 3D. Even though oMSCs from all donors differentiated, there was no clear profile in highly responsive donors between the three lineages. For example, a donor that was highly responsive during chondrogenic differentiation (donor 4), was not as responsive to osteogenic (donor 1) or adipogenic differentiation (donor 13). The variation or deviations among individuals is due to a single characteristic or several characteristics. Those differences which in their totality distinguish one individual from another include somatic or observed characteristics like physical, mental, social and cultural differences, as well as characteristics at the cellular and molecular levels. However, the variations of stem cells between individuals are still not fully explained. The relation between the performance of MSCs donors can be explained by the differences of the genetic epitopes and other proteins that are produced by the cells, or the proteins from their extracellular environments that affect the stem cell behaviour. The stemness of stem cells can be affected by the individual variation, which is affected by several factors including genetics. Hence, some individuals have the tendency to be obese, some have strong bones or strong muscles. Despite advances in stem cell biology, the behaviour of the stem cells is still not fully explained. Furthermore, adults stem cells are still not developed to their final lineage. Thus, cells can be variable according to their epitopes that they express, for example, the cells that we have isolated differed for STRO-4 epitopes on their cell membrane (STRO-4 positives and STRO-4 negative), even though they derived from the same bone marrow sample from the same donor at the same time. Additionally, the expression of epitopes may be affected by the environment and since cells have diversity in the response to different stimulants, this might also affect donor variability. A better understanding of functional properties through molecular profiling of MSCs may have impact on future clinical applications [49].

To monitor the chondrogenesis, the pellets were tested for the glycosaminoglycans increasing within the surrounding matrix. The DNA content of the pellets decreased with time in the chondrogenic medium (CM) treated group compared to the BM group. However, normalization of the sGAG to the pellet’s DNA content shows that sGAG synthesis increased over the culture period in the CM group as one would expect. Cell aggregates grown in the BM did not form cohesive pellets. Pellets remained fragile and broke easily. Whilst sGAG content remained high, possibly due to its incorporation in a stable ECM, the number of cells contributing to the sGAG may be underestimated whilst sGAG per cell becomes overestimated. Therefore, normalization of sGAG to DNA can effectively show whether the sGAG synthesis increases per cell. This has been shown to be a reliable measure when both DNA and GAG are increasing, or the DNA content is stable [50].

CD markers have been used to characterize stem cell populations with clear guidelines now in place for establishing genotype and phenotype of bone marrow derived mesenchymal stem cells [27]. However, it has not been concluded if MSCs resident in different tissues are the same or even very similar. For instance, adipose-derived MSCs express CD 34 [51], whereas BM-MSCs do not. W8-B2/MSCA-1 is expressed by BM-MSCs but not by placenta-derived MSCs [52]. In this study, the ovine BM-MSC expression for CD 29, CD 44, CD 45 and CD 31 was investigated. These antibodies were chosen because these epitopes are normally expressed positively (CD 29, CD 44) or negatively (CD 45, CD 31) in MSCs.

For the majority of the 13 donors, the CD marker expression confirmed this norm for ovine BM-MSCs. Cells expressed CD 29 (4 donors were +, 9 donors were ++) and CD 44 (1 donor was +, 11 donors were ++). On the other hand, negative expression was confirmed for CD 45 (11 donors were -, and 2 donors were ±) and CD 31(3 donors were -, 7 donors were ±, 3 donors were +). These results are consistent with a study by Boxall and Jones (2012) who scored MSC marker expression levels using the same scale [27].

Although bone marrow and adipose tissue are the main sources of the MSCs [13,52], perinatal sources, including amniotic membrane and umbilical cord have preference over adult sources due to availability, lack of donor site morbidity, young age of cells, high quantity of cells in the tissue, or high proliferation capacity [53] [54]. *In vitro* studies comparing MSCs from different sources concluded that MSCs are similar [55–57]. It is suggested that a better understanding of functional properties indicating the potential impact on future clinical applications may be achieved by molecular profiling of MSCs [58].

In summary, we have investigated the variability of MSCs derived from 13 sheep donors for their trilineage differentiation potential following selection of suitable differentiation media. Sheep are well accepted as pre-clinical models for orthopaedic tissue engineering studies, also, animal models for orthopaedic tissue engineering and disease modelling [59]. Testing of new purified and expanded MSC-based products in large animal models will allow for a thorough pre-clinical evaluation of novel products prior to clinical trials in humans [60].

## References

[1] Kumar S, Raj S, Kolanthai E, et al. Chemical functionalization of graphene to augment stem cell osteogenesis and inhibit biofilm formation on polymer composites for orthopedic applications. ACS Appl Mater Interfaces. 2015 Feb 11;7(5):3237–3252. 25584679 10.1021/am5079732

[2] Burt RK, Loh Y, Pearce W, et al. Clinical applications of blood-derived and marrow-derived stem cells for nonmalignant diseases. JAMA. 2008;299(8):925–936. DOI:10.1001/jama.299.8.925 18314435

[3] Cibelli J, Emborg ME, Prockop DJ, et al. Strategies for improving animal models for regenerative medicine. Cell Stem Cell. 2013 Mar 7;12(3):271–274. DOI:10.1016/j.stem.2013.01.004 23472868 PMC4383280

[4] Hulsart-Billström G, Dawson JI, Hofmann S, et al. A surprisingly poor correlation between in vitro and in vivo testing of biomaterials for bone regeneration: results of a multicentre analysis. Eur Cell Mater. 2016;24(31):312–322. 10.22203/ecm.v031a20 27215739

[5] Wilke HJ, Kettler A, Wenger KH, et al. Anatomy of the sheep spine and its comparison to the human spine. Anat Rec. 1997;247(4):542–555. 9096794 10.1002/(SICI)1097-0185(199704)247:4<542::AID-AR13>3.0.CO;2-P

[6] Egermann M, Goldhahn J, Holz R, et al. A sheep model for fracture treatment in osteoporosis: benefits of the model versus animal welfare. Lab Anim. 2008;42(4):453–464. 18782823 10.1258/la.2007.007001

[7] Hoemann C, Kandel R, Roberts S, et al. International cartilage repair society (ICRS) recommended guidelines for histological endpoints for cartilage repair studies in animal models and clinical trials. Cartilage. 2011;2(2):153–172. DOI:10.1177/1947603510397535 26069577 PMC4300784

[8] Music E, Futrega K, Doran MR. Sheep as a model for evaluating mesenchymal stem/stromal cell (MSC)-based chondral defect repair. Osteoarthritis Cartilage. 2018;26(6):730–740. 29580978 10.1016/j.joca.2018.03.006

[9] Yoshiya S, Dhawan A. Cartilage repair techniques in the knee: stem cell therapies. Curr Rev Musculoskelet Med. 2015;8(4):457–466. 26373771 10.1007/s12178-015-9302-y PMC4630225

[10] Moran CJ, Ramesh A, Brama PAJ, et al. The benefits and limitations of animal models for translational research in cartilage repair. J Exp Orthop. 2016 Jan 6;3(1):1. 26915001 10.1186/s40634-015-0037-x PMC4703594

[11] Adamzyk C, Emonds T, Falkenstein J, et al. Different culture media affect proliferation, surface epitope expression, and differentiation of ovine MSC. Stem Cells Int. 2013;2013. DOI:10.1155/2013/387324. PMC3819026 24228035

[12] Rhodes NP, Srivastava JK, Smith RF, et al. Heterogeneity in proliferative potential of ovine mesenchymal stem cell colonies. J Mater Sci. 2004;15(4):397–402. 10.1023/b:jmsm.0000021109.21807.f0 15332606

[13] Pittenger MF, Mackay AM, Beck SC, et al. Multilineage potential of adult human mesenchymal stem cells. Science. 1999;284(5411):143–147. DOI:10.1126/science.284.5411.143 10102814

[14] Buckwalter JA, Mankin HJ, Grodzinsky AJ. Articular Cartilage and Osteoarthritis. Instr Course Lect. 2005;54:465–480. 15952258

[15] Mikos AG, Herring SW, Ochareon P, et al. Engineering complex tissues. Tissue Eng. 2006;12(12):3307–3339. DOI:10.1089/ten.2006.12.3307 17518671 PMC2821210

[16] Redman SN, Oldfield SF, Archer CW. Current strategies for articular cartilage repair. Eur Cell Mater. 2005;9(23–32):23–32. 15830323 10.22203/ecm.v009a04

[17] Athanasiou KA, Zhu CF, Wang X, et al. Effects of aging and dietary restriction on the structural integrity of rat articular cartilage. Ann Biomed Eng. 2000;28(2):143–149. 10710185 10.1114/1.238

[18] Markides H, McLaren JS, Telling ND, et al. Translation of remote control regenerative technologies for bone repair. NPJ Regen Med. 2018;3(1):1–12. DOI:10.1038/s41536-018-0048-1 29675269 PMC5904134

[19] Strober W. Trypan blue exclusion test of cell viability. Curr Protoc Immunol. 1997;21(1):A. 3. 1–. 3. 2. 10.1002/0471142735.ima03bs21 18432654

[20] Bonnier F, Keating ME, Wrobel TP, et al. Cell viability assessment using the Alamar blue assay: a comparison of 2D and 3D cell culture models. Toxicol Vitro. 2015;29(1):124–131. DOI:10.1016/j.tiv.2014.09.014 25300790

[21] Crawford A, Frazer A, Lippitt JM, et al. A case of chondromatosis indicates a synovial stem cell aetiology. Rheumatology. 2006;45(12):1529–1533. 16670157 10.1093/rheumatology/kel111

[22] Osteogenic differentiation of purified, culture‐expanded human mesenchymal stem cells in vitro - Jaiswal - 1997 - Journal of Cellular Biochemistry - Wiley Online Library [Internet]. [cited 2021 Nov 15]. Available from: https://onlinelibrary.wiley.com/doi/abs/10.1002/SICI1097-464419970264:2%3C295:AID-JCB12%3E3.0.CO;2-I 9027589

[23] Heidari B, Shirazi A, Akhondi MM, et al. Comparison of proliferative and multilineage differentiation potential of sheep mesenchymal stem cells derived from bone marrow, liver, and adipose tissue. Avicenna J Med Biotechnol. 2013;5(2):104–117. DOI:10.1155/2012/123030 23799179 PMC3689554

[24] Jaiswal N, Haynesworth SE, Caplan AI, et al. Osteogenic differentiation of purified, culture-expanded human mesenchymal stem cells in vitro. J Cell Biochem. 1997;64(2):295–312. 9027589

[25] Jackson L, Jones DR, Scotting P, et al. Adult mesenchymal stem cells: differentiation potential and therapeutic applications. J Postgrad Med. 2007 Apr 1;53(2):121. 17495381 10.4103/0022-3859.32215

[26] McCarty RC, Gronthos S, Zannettino AC, et al. Characterisation and developmental potential of ovine bone marrow derived mesenchymal stem cells. J Cell Physiol. 2009;219(2):324–333. 19115243 10.1002/jcp.21670

[27] Boxall SA, Jones E. Markers for characterization of bone marrow multipotential stromal cells. Stem Cells Int. 2012 May 14;2012:e975871. DOI:10.1155/2012/975871. PMC3361338 22666272

[28] Berset CM, Lanker U, Zeiter S. Survey on sheep usage in biomedical research. Animals. 2020 Sep;10(9):1528. 32872575 10.3390/ani10091528 PMC7552153

[29] Jessop HL, Noble BS, Cryer A. The differentiation of a potential mesenchymal stem cell population within ovine bone marrow. Biochem Soc Trans. 1994 Aug 1;22(3):248S. 7821511 10.1042/bst022248s

[30] Kunisaki SM, Fuchs JR, Steigman SA, et al. A comparative analysis of cartilage engineered from different perinatal mesenchymal progenitor cells. Tissue Eng. 2007 Nov 1;13(11):2633–2644. 17655491 10.1089/ten.2006.0407

[31] Fadel L, Viana BR, Feitosa MLT, et al. Protocols for obtainment and isolation of two mesenchymal stem cell sources in sheep. Acta Cirúrgica Bras. 2011 Aug;26:267–273. 10.1590/s0102-86502011000400004 21808838

[32] Lyahyai J, Mediano DR, Ranera B, et al. Isolation and characterization of ovine mesenchymal stem cells derived from peripheral blood. BMC Vet Res. 2012 Sep 22;8(1):169. DOI:10.1186/1746-6148-8-169 22999337 PMC3514285

[33] Prolonged in vitro expansion partially affects phenotypic features and osteogenic potential of ovine amniotic fluid-derived mesenchymal stromal cells - ScienceDirect [Internet]. [cited 2021 Nov 15]. Available from: https://www.sciencedirect.com/science/article/abs/pii/S1465324913004933 10.1016/j.jcyt.2013.03.014 23768926

[34] Mrozik KM, Zilm PS, Bagley CJ, et al. Proteomic characterization of mesenchymal stem cell-like populations derived from ovine periodontal ligament, dental pulp, and bone marrow: analysis of differentially expressed proteins. Stem Cells Dev. 2010 Oct 1;19(10):1485–1499. DOI:10.1089/scd.2009.0446 20050811

[35] Godoy RF, Alves ALG, Gibson AJ, et al. Do progenitor cells from different tissue have the same phenotype? Res Vet Sci. 2014 Jun 1;96(3):454–459. 24636541 10.1016/j.rvsc.2014.02.013

[36] Cui P, He X, Pu Y, et al. Biological characterization and pluripotent identification of sheep dermis-derived mesenchymal stem/progenitor cells. BioMed Res Int. 2014 May 18;2014:e786234. 10.1155/2014/786234 PMC4052519 24949469

[37] Koobatian MT, Liang MS, Swartz DD, et al. Differential effects of culture senescence and mechanical stimulation on the proliferation and leiomyogenic differentiation of msc from different sources: implications for engineering vascular grafts. Tissue Eng Part A. 2015 Apr 1;21(7–8):1364–1375. 25517657 10.1089/ten.tea.2014.0535 PMC4394877

[38] Letouzey V, Tan KS, Deane JA, et al. Isolation and characterisation of mesenchymal stem/stromal cells in the ovine endometrium. PLoS ONE. 2015 May 18;10(5):e0127531. DOI:10.1371/journal.pone.0127531 25992577 PMC4436363

[39] Rentsch C, Hess R, Rentsch B, et al. Ovine bone marrow mesenchymal stem cells: isolation and characterization of the cells and their osteogenic differentiation potential on embroidered and surface-modified polycaprolactone-co-lactide scaffolds. Vitro Cell Dev Biol - Anim. 2010 Jul 1;46(7):624–634. DOI:10.1007/s11626-010-9316-0 20490706

[40] Delling U, Lindner K, Ribitsch I, et al. Comparison of bone marrow aspiration at the sternum and the tuber coxae in middle-aged horses. Can J Vet Res. 2012 Jan 1;76(1):52–56. 22754095 PMC3244288

[41] Vivas D, Caminal M, Oliver-Vila I, et al. Derivation of multipotent mesenchymal stromal cells from ovine bone marrow. Curr Protoc Stem Cell Biol. 2018;44(1): 2B.9.1-2B.9.22. DOI:10.1002/cpsc.43 29512111

[42] Risbud MV, Shapiro IM, Guttapalli A, et al. Osteogenic potential of adult human stem cells of the lumbar vertebral body and the iliac crest. Spine. 2006 Jan 1;31(1):83–89. DOI:10.1097/01.brs.0000193891.71672.e4 16395182

[43] Concise review: the surface markers and identity of human mesenchymal stem cells - Lv - 2014 - STEM CELLS. Wiley Online Lib. [cited 2021 Nov 15]. Available from. Internet. https://stemcellsjournals.onlinelibrary.wiley.com/doi/full/10.1002/stem.1681 10.1002/stem.1681 24578244

[44] Williams EL, White K, Oreffo ROC. Isolation and enrichment of Stro-1 immunoselected mesenchymal stem cells from adult human bone marrow. In: Turksen K editor. Stem Cell Niche: methods and Protocols [Internet]. Totowa, NJ: Humana Press; 2013pp. 67–73. cited 2021 Nov 15. Methods in Molecular BiologyAvailable from. DOI:10.1007/978-1-62703-508-8_7 23959983

[45] Gronthos S, McCarty R, Mrozik K, et al. Heat shock protein-90 beta is expressed at the surface of multipotential mesenchymal precursor cells: generation of a novel monoclonal antibody, STRO-4, with specificity for mesenchymal precursor cells from human and ovine tissues. Stem Cells Dev. 2009 Nov 1;18(9):1253–1262. DOI:10.1089/scd.2008.0400 19327008

[46] Substantial differences between human and ovine mesenchymal stem cells in response to osteogenic media: how to explain and how to manage? | BioResearch Open Acc. [cited 2021 Nov 15]. Available from. Internet. https://www.liebertpub.com/doi/full/10.1089/biores.2013.0029 10.1089/biores.2013.0029 PMC3776620 24083091

[47] Markides H, Newell KJ, Rudorf H, et al. Ex vivo MRI cell tracking of autologous mesenchymal stromal cells in an ovine osteochondral defect model. Stem Cell Res Ther. 2019 Jan 11;10(1):25. DOI:10.1186/s13287-018-1123-7 30635066 PMC6330448

[48] Mrugala D, Bony C, Neves N, et al. Phenotypic and functional characterisation of ovine mesenchymal stem cells: application to a cartilage defect model. Ann Rheum Dis. 2008;67(3):288–295. DOI:10.1136/ard.2007.076620 17644536

[49] Wegmeyer H, Bröske AM, Leddin M, et al. Mesenchymal stromal cell characteristics vary depending on their origin. Stem Cells Dev. 2013 Oct 1;22(19):2606–2618. 23676112 10.1089/scd.2013.0016 PMC3780294

[50] Dale TP Investigating the chondrogenic phenotype in clinically relevant cells: the effect of hTERT expression. [Internet] [doctoral]. Keele University. Keele University; 2016 [cited 2021 Nov 15]. Available from: https://eprints.keele.ac.uk/2440/

[51] Quirici N, Scavullo C, de Girolamo L, et al. Anti-L-NGFR and -CD34 monoclonal antibodies identify multipotent mesenchymal stem cells in human adipose tissue. Stem Cells Dev. 2010 Jun 1;19(6):915–925. DOI:10.1089/scd.2009.0408 19929314

[52] Battula VL, Bareiss PM, Treml S, et al. Human placenta and bone marrow derived MSC cultured in serum-free, b-FGF-containing medium express cell surface frizzled-9 and SSEA-4 and give rise to multilineage differentiation. Differentiation. 2007 Apr 1;75(4):279–291. DOI:10.1111/j.1432-0436.2006.00139.x 17288545

[53] Zuk PA, Zhu M, Ashjian P, et al. Human adipose tissue is a source of multipotent stem cells. Mol Biol Cell. 2002;13(12):4279–4295. DOI:10.1091/mbc.e02-02-0105 12475952 PMC138633

[54] Ilancheran S, Moodley Y, Manuelpillai U. Human fetal membranes: a source of stem cells for tissue regeneration and repair? Placenta. 2009;30(1):2–10. 18995896 10.1016/j.placenta.2008.09.009

[55] Baksh D, Yao R, Tuan RS. Comparison of proliferative and multilineage differentiation potential of human mesenchymal stem cells derived from umbilical cord and bone marrow. Stem Cells. 2007;25(6):1384–1392. 17332507 10.1634/stemcells.2006-0709

[56] Barlow S, Brooke G, Chatterjee K, et al. Comparison of human placenta-and bone marrow–derived multipotent mesenchymal stem cells. Stem Cells Dev. 2008;17(6):1095–1108. DOI:10.1089/scd.2007.0154 19006451

[57] Kern S, Eichler H, Stoeve J, et al. Comparative analysis of mesenchymal stem cells from bone marrow, umbilical cord blood, or adipose tissue. Stem Cells. 2006;24(5):1294–1301. 16410387 10.1634/stemcells.2005-0342

[58] Wegmeyer H, Bröske AM, Leddin M, et al. Mesenchymal stromal cell characteristics vary depending on their origin. Stem Cells Dev. 2013;22(19):2606–2618. DOI:10.1089/scd.2013.0016 23676112 PMC3780294

[59] McGovern JA, Griffin M, Hutmacher DW. Animal models for bone tissue engineering and modelling disease. Dis Model Mech. 2018;11(4):dmm033084. 29685995 10.1242/dmm.033084 PMC5963860

[60] Bieback K, Kinzebach S, Karagianni M. Translating research into clinical scale manufacturing of mesenchymal stromal cells. Stem Cells Int. 2011;2010. DOI:10.4061/2010/193519 PMC3034974 21318154

